# Infectious Diseases

**Published:** 1893-04

**Authors:** W. G. Eggleston


### INFECTIOUS DISEASES.

The term "infectious disease" is a very general one, and for the purpose of this article I shall include under it all communicable diseases—all diseases that can be in any way communicated to one person from another. These diseases are: Asiatic cholera, yellow fever, small-pox, diphtheria, scarlet fever, measles, typhus fever, typhoid fever, whooping cough and a few others.

So far as the treatment of a particular case of one of these diseases is concerned, that must be left to the attending physician. Any attempt to tell even the most enlightened part of the public how to deal with a case of one of these diseases could result only in imparting the little knowledge that is so dangerous. The most that can be said, therefore, is that patients suffering with these diseases must have plenty of palatable easily digested food, pure water and fresh air, and must be kept clean.

So far as the public is concerned, the most important part of the treatment of communicable diseases consists in preventing their spread from the sick persons to others.

Some diseases that were once thought not communicable have been found in recent years to own their chief danger to the fact that they are infectious—communicable. This is true of consumption, which is the most dangerous of all diseases in the sense that it causes more deaths than any other disease.

If we could only vaccinate against all other preventable diseases as we can against smallpox, it would be a comparatively easy matter to stamp them out and prevent them; but they are spread in different ways, and the methods of preventing them are different. In each disease something goes from a sick person. This "something" is capable of causing the disease in other persons.

The actual cause of consumption, so far as we know now, goes out in the sputum (spittle, expectoration), and is thus scattered where the moist sputum goes, as well as by the dust after the sputum dries. The first step, then, in preventing consumption is to destroy or disinfect all the sputum from each consumptive.

The germs of consumption may be in food or drink. The milk and flesh of consumptive cows and other animals often contain the germs of the disease. Neither the flesh nor the milk of such animals should be used for food.

Not less important than destroying or disinfecting the sputum of consumptives are fresh air and cleanliness. People who live in unclean and ill-ventilated houses or apartments are in a fair way to absorb and retain the germs of consumption. It has been suggested by some sanitarians that healthy persons should not sleep in the same bed nor in the same room with a person that has consumption. It is the part of prudence to comply with this suggestion, since it is a reasonable precaution.

There should never be a public or church funeral of any person dead of cholera, small-pox, typhus fever, diphtheria, yellow fever, scarlet fever or measles. The corpses of such persons should be buried as quickly as possible, and should never be transported in a railway train or other public vehicle. Public safety demands that all such corpses be wrapped immediately after death in a sheet thoroughly wetted with a solution of corrosive sublimate (half an ounce to two gallons of water), and the coffin then closed immediately and permanently. Funeral services should not be held in the same room with the body.

All this may seem a harsh way of dealing with the sacred clay of those we love, but we cannot get away from the fact that the safety of the living should be our first consideration. Necessary prudence does not imply disrespect to the dead. And surely none of us would wish to be, when dead, the means of bringing illness and death to the living.

I have said nothing of the advances made of late years in treating individual cases of these diseases, because the real progress has been in the way of prevention. The individual case must be treated by the physician, but the higher work of prevention cannot be carried on without the hearty co-operation of fathers and mothers—of all the people in the community.

Every one can do something. Every householder can help by promptly reporting the fact when any infectious disease breaks out in his or her house, by warning his neighbors of it so that they and their children shall not be exposed to the danger.

Many people have a foolish objection to having an infectious disease placard on their houses. The objection is not only foolish, but it shows a disregard of the rights of other people. It is a crime to be the means direct or indirect, of exposing others to unnecessary danger.

As the treatment of cases of illness costs money, so the efficient prevention of disease must cost money. But prevention costs less than treatment in the long run. The efficiency of a health officer is not to be measured by the number of epidemics that he stamps out, but by the absence of epidemics.

If he keeps his town in such a sanitary healthy condition that infectious diseases do not occur, he is worth ten times the money paid to him. If each village and city were to pay annually for a health organization as much money as is spent for the fire or police department, the money would be invested at a high rate of interest.

The three greatest advances in the treatment of infectious diseases are disinfectants, the health officer and prevention.

W. G. Eggleston, M. D.

